# Long-Term (10-Year) Gastrointestinal and Genitourinary Toxicity after Treatment with External Beam Radiotherapy, Radical Prostatectomy, or Brachytherapy for Prostate Cancer

**DOI:** 10.1155/2012/853487

**Published:** 2012-04-11

**Authors:** Grant K. Hunter, Chandana A. Reddy, Eric A. Klein, Patrick Kupelian, Kenneth Angermeier, James Ulchaker, Nabil Chehade, Andrew Altman, Jay P. Ciezki

**Affiliations:** ^1^Cleveland Clinic Department of Radiation Oncology, 9500 Euclid Avenue, Cleveland, OH 44195, USA; ^2^Cleveland Clinic GlickmanUrological and Kidney Institute, 9500 Euclid Avenue, Cleveland, OH 44195, USA; ^3^UCLA Jonsson Comprehensive Cancer Center, 200 UCLA Medical Plaza, Suite B265, Los Angeles, CA 90095-6951, USA; ^4^Department of Urology, Kaiser Permanente, Ohio, 12301 Snow Road, Parma, OH 44130, USA

## Abstract

*Objective.*To examine gastrointestinal (GI) and genitourinary (GU) toxicity profiles of patients treated in 1999 with external beam radiotherapy (RT), prostate interstitial brachytherapy (PI) or radical prostatectomy (RP). *Methods.* TThe records of 525 patients treated in 1999 were reviewed to evaluate toxicity. Late GI and GU morbidities were graded according to the RTOG late morbidity criteria. Other factors examined were patient age, BMI, smoking history, and medical co-morbidities. Due to the low event rate for late GU and GI toxicities, a competing risk regression (CRR) analysis was done with death as the competing event. *Results.* Median follow-up time was 8.5 years. On CRR univariate analysis, only the presence of DM was significantly associated with GU toxicity grade >2 (*P* = 0.43, HR 2.35, 95% Cl = 1.03–5.39). On univariate analysis, RT and DM were significantly associated with late GI toxicity. On multivariable analysis, both variables remained significant (RT: *P* = 0.038, HR = 4.71, CI = 1.09–20.3; DM: *P* = 0.008, HR = 3.81, 95% Cl = 1.42–10.2). *Conclusions.* Late effects occur with all treatment modalities. The presence of DM at the time of treatment was significantly associated with worse late GI and GU toxicity. RT was significantly associated with worse late GI toxicity compared to PI and RP.

## 1. Introduction

For patients with localized prostate cancer, treatment options include active surveillance, radical prostatectomy (RP), external beam radiation therapy (RT), or low-dose-rate prostate interstitial brachytherapy (PI). There is data showing that in low-risk disease, excellent prostate-specific antigen (PSA) relapse-free survival outcomes are obtained with a low risk of significant treatment-related morbidity [1–4]. Low-risk patients do well with any of the three treatment modalities but there is no data comparing late toxicity. Due to lack of randomized data comparing these modalities, treatment decisions are often made by patient or physician preference based on the side effect profiles exhibited by each treatment type. While all modalities have generally similar toxicity profiles, there are slight differences between each that can impact long-term quality of life [5]. There are reports comparing the late toxicity rates between different types of radiation treatment options with noted improvement in morbidity with newer treatment techniques such as intensity-modulated radiation therapy (IMRT) [6]. However, there have been few reports comparing potential long-term toxicity profiles of patients treated with radiation treatment modalities to those who have undergone surgical resection. In the present paper we compare our data on toxicity for patients treated in 1999 with RP, RT (IMRT), or PI. 

## 2. Materials and Methods

The records of 525 patients treated in 1999 were reviewed to evaluate toxicity profiles. The year 1999 was chosen to allow for sufficient potential follow-up time, and by that time, all the three treatment programs (RP, RT, and PI) had been well established for years. Of all patients treated during the 1999 calendar year we excluded 42 patients for whom we had no follow-up data, with the excluded patients being evenly distributed among the three methods of treatment. All patients were identified from our prospectively maintained, IRB-approved prostate cancer registry. Of the 483 patients with clinical followup, 116 (24%) were treated with PI, 195 (40%) with RP, and 172 (36%) with RT.

Each patient's medical record was examined with attention to visits to his urologist, radiation oncologist, internal medicine, or family care physician and any gastroenterologist since the time of treatment. Late toxicity was defined as toxicity occurring at least six months after treatment. Gastrointestinal (GI) and genitourinary (GU) morbidity profiles were specifically examined and graded according to the RTOG acute and late morbidity scoring criteria. The RTOG morbidity scoring criteria is a numeric scoring system with scores ranging from grade 1 (mild morbidity not requiring any intervention) to grade 5 (death), with specific criteria for GI and GU side effect profiles. These criteria were not effective in describing toxicities experienced by the group of patients treated with surgical resection, so the National Cancer Institute's Common Terminology Criteria for Adverse Events, (CTCAE) version 4.0, was used to grade surgical patients' late morbidity. The CTCAE is comparable, but not identical to the RTOG late toxicity scoring system. The CTCAE is generally more descriptive and comprehensive compared to the RTOG late morbidity scoring criteria; however there is significant overlap in the described grading systems. For example, the RTOG criteria donot mention a surgical intervention in their late GI scoring criteria save in the case of grade 3. However, the RTOG grade 2 late GI scoring criteria include: “moderate diarrhea and colic; bowel movement >5 times daily; excessive rectal mucus or intermittent bleeding.” When patients presented for followup with intermittent bleeding after RT we felt this was clearly a grade 2 toxicity and not a grade 3, which the RTOG criteria list as being: “obstruction or bleeding requiring surgery.” This description of late GI toxicity that includes intermittent bleeding is similar to the CTCAE grade 2 GI toxicity which is listed under rectal hemorrhage as: “moderate symptoms; medical intervention or minor cauterization indicated.” The late GU toxicity criteria are also slightly different as the RTOG criteria never mention surgical intervention at all, and late grade 2 toxicity incorporates: “moderate frequency; generalized telangiectasia; intermittent macroscopic hematuria.” The CTCAE grade 2 hematuria is listed as including “symptomatic, urinary catheter or bladder irrigation indicated; limiting instrumental ADL.” Again, the scoring systems are comparable, but not identical.

Upon review of the patient's medical record, at any visit wherein toxicity was addressed or brought up by the patient, the specific GI or GU toxicity was graded according to the above criteria. Other factors examined were patient age, body mass index (BMI), smoking history, and medical co-morbidities including presence of diabetes mellitus (DM), peripheral vascular disease, and connective tissue disease.

Due to the low event rate and the potential for patients to have died before developing a late GU or GI toxicity, a competing risk regression (CRR) analysis was done for each toxicity endpoint with death as the competing event [7]. The final multivariable model was built using the forward, stepwise procedure. Factors with a *P* value < 0.05 on a univariate analysis were included in a multivariable analysis. Variables having a *P* value of < 0.05 remained in the final model. Cumulative incidence rates of late GU and GI were calculated, and comparisons among the three treatment groups were made using Gray's test [8]. The competing risk regression analysis and the cumulative incidence rate comparisons were performed using R version 2.8.1 (The R Foundation for Statistical Computing, Vienna, Austria). All other statistical analyses were done using SAS version 9.2 (SAS Institute, Cary, NC, USA).

### 2.1. Radical Prostatectomy Group

Patients in the surgical group underwent RP under the care of surgeons in the department of urology. The majority underwent open, retropubic radical prostatectomies. Twelve (6.2%) patients underwent a laparoscopic prostatectomy or robotic-assisted prostatectomy. Ninety-eight (50.3%) patients underwent a bilateral nerve sparing prostatectomy, 20 (10.3%) had unilateral nerve sparing, and 77 (39.5%) did not undergo a nerve-sparing prostatectomy. Twenty-three patients (11.8%) received adjuvant salvage radiation therapy to the prostate.

### 2.2. External Beam Radiation Therapy Group

All but 11 (6.4%) patients in the EBRT group were treated with IMRT. The patients were treated under the care of two radiation oncologists with the general treatment planning guidelines previously described [9, 10]. In brief, the patients were treated with a five-field IMRT plan to a total dose of 70 Gy via 28 daily fractions of 2.5 Gy. This dose and hypofractionated regimen was the standard at the Cleveland Clinic in 1999. Hypofractionated IMRT is one method of delivering external beam radiotherapy. It remains under investigation, with a phase III trial (RTOG 0415) conducted by the RTOG, with long-term data maturing. The 11 patients who were not treated with IMRT were treated with a conformal radiation treatment plan to a total dose of 78 Gy via 39 daily fractions of 2.0 Gy.

The treating physician delineated all target and normal tissue volumes, with low-risk patients having the target defined as the prostate only and high-risk patients having the prostate and seminal vesicles included as the target. The expansions to obtain a planning target volume (PTV) from the prostate or prostate + seminal vesicles were 4 mm posteriorly, 8 mm laterally, and 5 mm in all other dimensions. The dose constraints used for treatment planning included the target volume (PTV) to 70 Gy with a range of 65 to 78 Gy with the dose prescribed to an isodose line covering the target ranging from 82.0% to 90.0%. The limits that were used for bladder were no more than 30% to receive greater than 55 Gy with maximum level at 74 Gy and no more than 30% of rectum to be greater than 50 Gy with maximum dose of 74 Gy.

For daily localization, after set-up via triangulation marks, all patients underwent ultrasound image guidance using the BAT transabdominal ultrasound system. The system consists of a B-mode transabdominal ultrasound probe attached to a precision tracking arm with greater details described previously [9]. 

### 2.3. Prostate Brachytherapy Group

Patients in the brachytherapy group underwent PI under the care of one of two radiation oncologists. The brachytherapy procedure has been described previously [11]. In brief, patients were treated with a dose of 144 Gy prescribed to the prostate using I-125 monotherapy. All patients underwent intraoperative planning using a transrectal ultrasound probe for creation of an optimal seed loading pattern that would allow for delivery of the prescription dose and conform to established dose constraints for the surrounding normal tissues. The target volume was defined on the intraoperative ultrasound axial images and consisted of the prostate only for low-risk patients and the prostate and 0.5–1 cm of the proximal seminal vesicles for intermediate- and high-risk patients. A postprocedure CT scan was performed one month following implantation for the purpose of dosimetric evaluation. Dosimetric guidelines followed for evaluation of the brachytherapy consisted of minimum dose to 90% of the prostate PTV (D90) between 90% and 130% of the prescribed dose, at least 80% of the prostate PTV to get at least 100% of the prescribed dose, and the urethral dose should be limited to 150% of the target dose. The dosimetric constraint for the rectum was defined as the volume of rectum receiving 100% of the prescribed dose and it was limited to be less than 1 cc.

Follow-up evaluations were done one month after treatment for all three modalities. Subsequent follow-up examinations were done between three to six months and then yearly for five years. Following five years, followup with either the treating urologist or radiation oncologist was sporadic; however followup with the primary care physician was reviewed and coded according to the above-mentioned toxicity criteria. Most often, if any GI or GU events occurred and the patient was not currently following with his radiation oncologist or urologist, the patient was referred back for followup upon incidence of the toxicity. The median followup time for all groups of patients was 8.5 years (range 0.1–11.5 years). The median follow-up times for each group are as follows: RP: 8.6 years, PI: 8.1 years, EBRT: 8.9 years. The patient characteristics of all treatment groups are shown in Table 1.

## 3. Results

**Figure 1:**
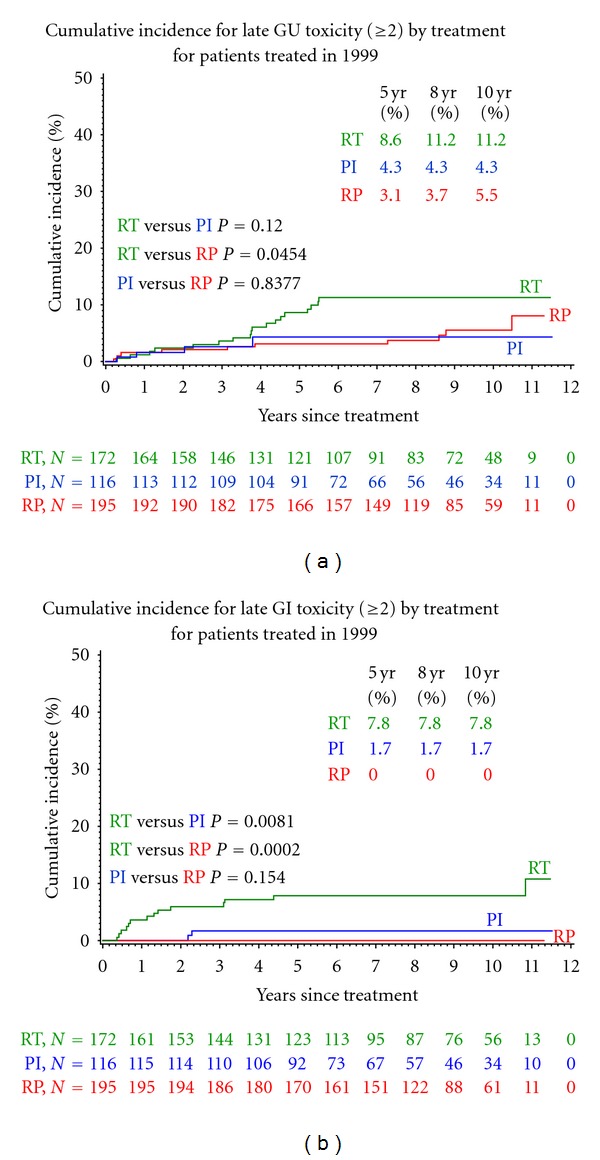
(a) Cumulative incidence curves of GU toxicity, ≥grade 2 for patients treated in 1999 with external beam radiation therapy, radical prostatectomy, or prostate brachytherapy. (b) Cumulative incidence curves of GI toxicity, ≥grade 2 for patients treated in 1999 with either external beam radiation therapy, radical prostatectomy, or prostate brachytherapy.

Overall, all three modalities were associated with relatively low late GI and GU toxicity rates. The frequency of late GU toxicity (grade 2 or higher) was 6.8% for all patients and was 4.3% for PI patients, 5.1% for RP patients, and 10.5% for RT patients. As demonstrated in Figure 1(a), the cumulative incidence of long-term GU toxicity (grade 2 or higher) was highest in patients treated with RT (11.2% at 10 years). Patients treated with RP had the next highest cumulative incidence of GU toxicity, 5.5% at 10 years, while the cumulative incidence at 10 years was 4.3% for PI. There were no GU toxicities noted beyond four years for PI patients. GU toxicities were observed more than eight years after treatment for RP patients. The varying types of late toxicities were slightly different following RP compared to those after PI or RT. The slight variations as well as the relative frequency are noted in Table 2. 

In the competing risk regression univariate analysis, the presence of DM was associated with higher rates of GU toxicity (*P* = 0.043, HR 2.35, 95% CI = 1.03–5.39, Table 3(a)). DM was the only factor that was statistically significant with relation to the onset of late GU toxicity. Type of treatment was not significantly associated with late GU toxicity. The major causes of GU toxicity were increased frequency/irritation and varying degrees of incontinence. The vast majority of toxicities resolved with further followup or intervention. 

There were no late GI toxicities (grade 2 or higher) observed for patients who underwent RP (Figure 1(b)). The frequency of late GI toxcitiy (grade 2 or higher) was 3.3% for all other patients. For patients treated with PI the frequency was 1.7% and was 8.1% for patients treated with RT. The 10-year cumulative incidence rate of late GI toxicity was 7.8% in patients who had RT versus 1.7% for PI patients.

Since there were no events in the RP group, only the PI and RT patients were included in the CRR analysis for late GI toxicity grade ≥2. On univariate analysis, RT and DM were significantly associated with late GI toxicity. On multivariable analysis, both variables remained significant (RT: *P* = 0.038, HR = 4.71, 95% CI = 1.09–20.3; DM: *P* = 0.008, HR = 3.81, 95% CI = 1.42–10.2; Table 3(b)). The major causes of GI toxicity were rectal bleeding and proctitis. The vast majority of toxicities resolved with further followup or intervention.

## 4. Comment

Our results show that patients treated with any of the three modalities had low rates of late toxicity. The median follow-up time of 103 months is longer than most retrospective series or cohort studies examining the toxicities associated with prostate cancer treatments. We appreciated an increase in both GU and GI long-term toxicity, in patients with pre-existing DM. We also observed an increased incidence of long-term GI toxicity in patients treated with RT relative to patients treated with RP or PI that was significant on univariate and multivariable analysis. Our data confirmed that long-term GI toxicity is lower in RP patients compared with either modality of radiation treatment which correlates with data from multiple quality-of-life studies [12–14].

Diabetes has been shown to be a known risk factor for worse long-term toxicities after treatment for prostate cancer. In the CaPSURE study, a longitudinal disease registry of men with prostate cancer, men with DM who were treated with radical prostatectomy had worse urinary control over a follow-up period of two years [15]. Men with DM have been shown to have worse late GI and GU side effects after being treated with 3D conformal radiation therapy compared to nondiabetic men [16, 17]. In a series from the British Columbia Cancer Agency, men treated with I-125 brachytherapy and who had DM were more likely to have late (median followup 57 months) symptomatic flares of GU toxicity as measured by the International Prostate Symptom Score (IPSS) [18].

This association of DM with worse long-term morbidity correlates with a report from Thong et al. [19]. They examined the data from the Prostate Cancer Outcomes Study (PCOS) with specific attention to the longitudinal effect of DM on the health-related quality of life (HRQOL) measures that were collected. With five years of followup, they reported that men with DM who were diagnosed prior to treatment had worse HRQOL scores across treatment groups of radical prostatectomy, radiation therapy, androgen deprivation, or watchful waiting. Furthermore, diabetic men had the lowest urinary control and sexual function scores over time. These differences in HRQOL remained constant over the five years evaluated. There are several reports that show the presence of DM leads to an increase in all-cause mortality among patients with prostate cancer, but not an increase in prostate-cancer-related death, suggesting similar treatment efficacy [20–24]. Our data further adds that late toxicity is seen more often in patients with DM compared to those without DM.

Other quality-of-life data that compares all three treatment modalities most often lack prolonged followup. The PROST-QA trial had 24 months of followup and demonstrated the resolution of acute morbidity but did not describe further late toxicity [5]. In contrast with a study done by Miller et al. that was reported in 2005, our series of patients shows evolving long-term toxicity with all three types of treatment modalities [25]. Miller reported on patients who had received 3D-CRT, PI, or RP, with median time of followup ranging from 5.4 to 6.5 years since treatment and noted that patients who had undergone RP were unlikely to have further toxicity after a period of two years. Patients in this cohort who underwent RT or PI were more likely to have long-term emerging toxicity as reported by their patient-reported quality-of-life outcomes study. Our data, with median follow-up time ranging from 8.1 to 8.9 years since treatment, contradicts this finding as we found in our series that the cumulative incidence of GU toxicity for RP patients increased over time, even after five years. While this finding was novel, it is important to note that the cumulative rate was still low (5.5% at 10 years), and this may be noted in the cohort reported by Miller with continued followup.

The relative increase in GI toxicity seen in patients who underwent RT compared to RP and PI could potentially be attributed to the hypofractionated dose of their IMRT. In 1999, when these treatments were performed, the institutional practice at the Cleveland Clinic was to treat prostate cancer via hypofractionated IMRT at 2.5 Gy/fraction. With the slightly larger dose per fraction compared to more traditional dosing (1.8–2.0 Gy/fraction), it could be hypothesized the late effects are results of the 2.5 Gy/fraction regimen as fraction size is the dominant factor in determining late effects from radiotherapy [26]. Earlier reports of the tolerability of the hypofractionated regimen show it to be comparable to more standard dose fractionation schemes with regard to disease control and toxicity rates [27, 28]. These data are a continuation of followup of a part of the patient cohort described in the reports by Kupelian et al. and could simply show that with longer followup late GI toxicity will occur [28]. However, it is important to note that the cumulative incidence rate for this toxicity is still low. In a randomized trial published recently by Yeoh et al. patients treated with hypofractionated radiation (55 Gy/20 fractions) compared to conventionally fractionated radiation (64 Gy/32 fractions) had equivalent rates of GI and GU toxicity with prospective toxicity data accrued up to 60 months after treatment, suggesting further that hypofractionated radiation treatment has similar toxicity to conventional radiation therapy [29].

While this study lacks the thorough, regimental followup of prospective clinical trials, it does capture the sort of toxicity that will cause a patient to be referred for intervention (e.g., urinary strictures requiring dilation and rectal bleeding requiring cauterization). As such, this methodology will not adequately assess the subjective quality-of-life changes that equate to a grade I toxicity. It does, however, record the level of toxicity necessitating intervention and therefore gives a good estimate of the long-term cost in terms of objective quality-of-life changes as well as the cost in terms of medical resources used to deal with the sequelae of treatment. Further, since it is a retrospective study with physician-reported data there is a potential to underestimate the level of toxicity assessed. Patient-reported, prospective data would have been ideal but was not available. Our study is limited by the lack of follow-up data on sexual potency, which is an important factor in overall quality of life following treatment for prostate cancer.

## 5. Conclusion

Prostate cancer treatment is associated with low-level long-term GI and GU toxicity. While the incidence is small, the differences among modalities are significant. When assessing the effect of treatment, one must not only account for the cost of initial treatment, but also the cost of handling toxicity. The presence of diabetes adds to the risk of developing long-term toxicity.

## Figures and Tables

**Table 1:** Patient Characteristics.

Characteristic	RP (*n* = 195)	RT (*n* = 172)	PI (*n* = 116)	*P* value
Median age (yrs), (range)	62, (42–75)	68, (48–84)	69, (47–78)	<0.0001
Median follow-up time (yrs), (range)	8.6, (1.7–11.2)	8.9, (0.13–11.4)	8.1, (0.16–11.5)	0.08
Median body mass index (m/kg^2^), (range)	26.92, (19–39)	27.57, (18–42)	27.2, (20–42)	0.07
Ever smoked (%)	65.1	52.9	63.8	0.10
Diabetic (%)	6.7	15.1	10.3	0.02
Peripheral vascular disease (%)	1.5	3.5	7.8	0.02
Connective tissue disease (%)	0.5	0	0	0.48
Mean charlson score (range)	0.3, (0–3)	0.5, (0–7)	0.8, (0–7)	<0.0001
Androgen deprivation (%)	16.9	63.4	30.2	<0.0001
Risk category (%)				<0.0001
Low	50.8	30.8	75	
Intermediate	25.1	29.7	25	
High	24.1	39.5	0	

**Table 2:** Distribution of Toxicities Requiring Interventions (Corresponding to grade ≥2).

Modality most common toxicities	GU toxicities no. (%)	Modality most common toxicities	GI toxicities no. (%)
RP			
Urinary stricture	6 (3.0%)		
Incontinence	2 (1.0%)		
Urinary leakage	2 (1.0%)		
PI		PI	
Urinary stricture	4 (3.4%)	Rectal bleeding	2 (1.7%)
Incontinence	1 (0.8%)		
Retention	1 (0.8%)		
RT		RT	
Frequency	5 (2.9%)	Rectal bleeding	8 (4.7%)
Hematuria	7 (4.0%)	Radiation Proctitis	4 (2.3%)
Retention	6 (3.5%)	Increased freq.	2 (1.1%)

**Table:** (a)

Univariate analysis	*P* value	Hazard ratio	95% CI
Treatment			
RP versus PI	0.73	1.21	0.41–3.55
RT versus PI	0.07	2.54	0.94–6.87
RT versus RP	0.06	2.11	0.97–4.59
Age (continuous variable)	0.12	1.04	0.99–1.10
Charlson score	0.52	1.09	0.84–1.42
Peripheral vascular disease (yes versus no)	0.42	1.81	0.43–7.62
Diabetes (yes versus no)	0.043	2.35	1.03–5.39
Androgen deprivation (yes versus no)	0.65	0.85	0.41–1.74
Body mass index	0.40	1.03	0.97–1.09

**Table:** (b)

Univariate analysis	*P* value	Hazard ratio	95% CI
Treatment			
EBRT versus BT	0.03	5.10	1.17–22.3
Age (continuous variable)	0.27	1.05	0.96–1.14
Charlson score	0.13	1.21	0.94–1.56
Peripheral vascular disease (yes versus no)	0.85	1.22	0.17–9
Diabetes (yes versus no)	0.0051	4.2	1.54–11.5
Androgen deprivation (yes versus no)	0.63	0.78	0.29–2.11
Body mass index	0.88	0.99	0.83–1.17
Multivariable analysis			
Diabetes (yes versus no)	0.008	3.81	1.42–10.2
EBRT versus BT	0.038	4.71	1.09–20.3
